# 3-(1-Naphth­yl)-*N*-phenyl­oxirane-2-carboxamide

**DOI:** 10.1107/S1600536809048752

**Published:** 2009-11-21

**Authors:** Lian-Mei Chen, Tai-Ran Kang

**Affiliations:** a College of Chemistry and Chemical Engineering, China West Normal University, Nanchong 637002, People’s Republic of China

## Abstract

In the title compound, C_19_H_15_NO_2_, the mol­ecule adopts a *syn* configuration with the naphthalene and *N*-phenyl­formamide units located on the same side of the ep­oxy ring. The ep­oxy ring makes dihedral angles of 58.73 (9) and 65.18 (9)°, respectively, with the naphthalene ring system and the benzene ring. Inter­molecular N—H⋯O and C—H⋯O hydrogen bonding is present in the crystal structure.

## Related literature

For background to the use of epoxide-containing compounds as building blocks in the synthesis of biologically active compounds, see: Porter & Skidmore (2000 ▶); Shing *et al.* (2006 ▶); Watanabe *et al.* (1998 ▶). For related structures, see: He (2009 ▶); He & Chen (2009 ▶); He *et al.* (2009 ▶).
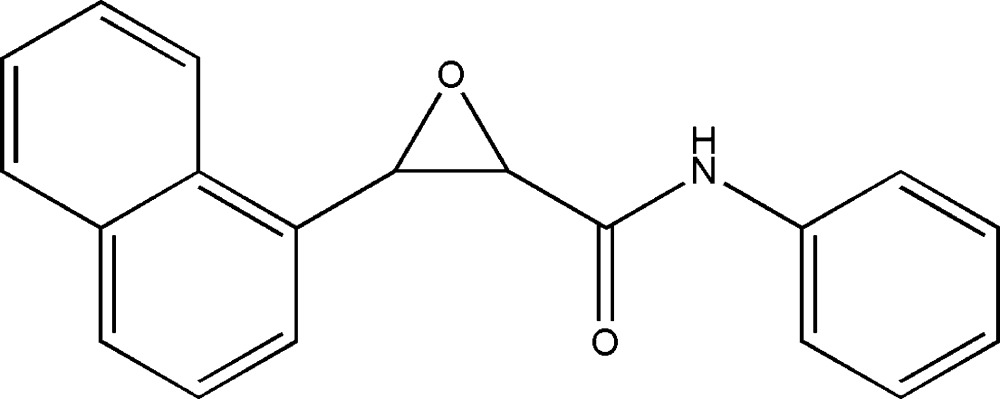


## Experimental

### 

#### Crystal data

C_19_H_15_NO_2_
*M* *_r_* = 289.32
Orthorhombic, 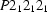

*a* = 6.62890 (10) Å
*b* = 10.03500 (10) Å
*c* = 23.2033 (3) Å
*V* = 1543.51 (3) Å^3^
*Z* = 4
Cu *K*α radiation
μ = 0.65 mm^−1^
*T* = 290 K
0.40 × 0.36 × 0.30 mm

#### Data collection

Oxford Diffraction Gemini S Ultra diffractometer
Absorption correction: multi-scan (*CrysAlis Pro*; Oxford Diffraction, 2009 ▶) *T*_min_ = 0.782, *T*_max_ = 0.829
13761 measured reflections
1783 independent reflections
1641 reflections with *I* > 2σ(*I*)
*R*_int_ = 0.031

#### Refinement

*R*[*F* ^2^ > 2σ(*F* ^2^)] = 0.032
*wR*(*F* ^2^) = 0.093
*S* = 1.08
1783 reflections
203 parameters
H atoms treated by a mixture of independent and constrained refinement
Δρ_max_ = 0.08 e Å^−3^
Δρ_min_ = −0.13 e Å^−3^

### 

Data collection: *CrysAlis Pro* (Oxford Diffraction, 2009 ▶); cell refinement: *CrysAlis Pro*; data reduction: *CrysAlis Pro*; program(s) used to solve structure: *SHELXS97* (Sheldrick, 2008 ▶); program(s) used to refine structure: *SHELXL97* (Sheldrick, 2008 ▶); molecular graphics: *ORTEP-3* (Farrugia, 1997 ▶); software used to prepare material for publication: *SHELXL97*.

## Supplementary Material

Crystal structure: contains datablocks global, I. DOI: 10.1107/S1600536809048752/xu2678sup1.cif

Structure factors: contains datablocks I. DOI: 10.1107/S1600536809048752/xu2678Isup2.hkl

Additional supplementary materials:  crystallographic information; 3D view; checkCIF report

## Figures and Tables

**Table 1:** Hydrogen-bond geometry (Å, °)

*D*—H⋯*A*	*D*—H	H⋯*A*	*D*⋯*A*	*D*—H⋯*A*
N1—H4⋯O2^i^	0.84 (2)	2.17 (2)	2.954 (1)	155.7 (18)
C5—H5⋯O1^ii^	0.93	2.58	3.431 (2)	153 (1)

Symmetry codes: (i) 
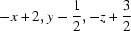
; (ii) 
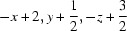
.

**Fig. 1.**
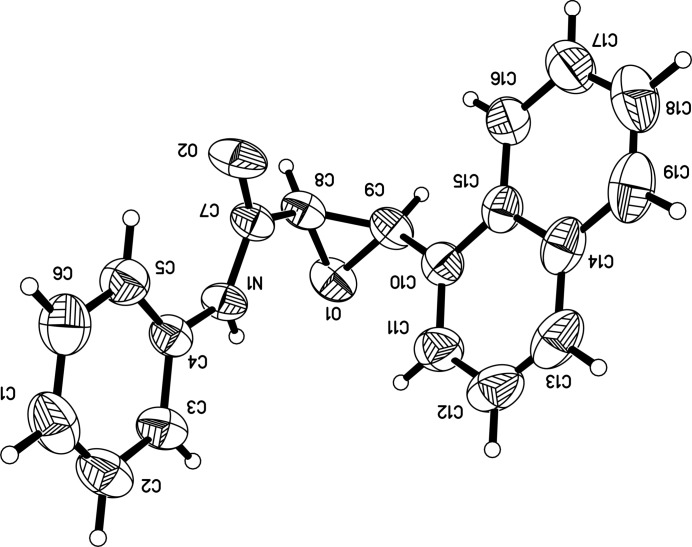
The molecular structure of (I) with 30% probability displacement ellipsoids (arbitrary spheres for H atoms).

## supplementary crystallographic information

### Comment

α,β-Epoxides are very important building blocks for the synthesis of complex molecules, in particular, of biologically active compounds (Porter & Skidmore, 2000; Shing *et al.*, 2006; Watanabe *et al.*, 1998). Various effective systems have been developed over the years for the preparation of α,β-epoxides. The most common approach to access these molecules is the epoxidation of α,β-unsaturatd carbonyl compound. As a part of our interest in the synthsis of epoxides ring systems, we synthesis the title compound by using Darzens reaction. We report herein the crystal structure of the title compound.

The molecular structure of (I) is shown in Fig. 1. Bond lengths and angles in (I) are normal. In the molecular, the 1-naphthyl ring with the phenyl ring adopts a *cis* configuration about the epoxides ring. The dihedral angle between the phenyl ring and the 1-naphthyl ring is 77.79 (4)°, O1/C8/C9 epoxide ring makes dihedral angles of 58.73 (9)° and 65.18 (9)° with the 1-naphthyl ring and phenyl ring, respectively. These values are very similar to those observed in related structures (He, 2009; He & Chen, 2009; He *et al.*, 2009). The crystal packing is stabilized by N—H···0 and C—H···0 hydrogen bonding (Table 1).

### Experimental

2-Chloro-*N*-phenylacetamide (0.085 g, 0.5 mmol) and potassium hydroxide (0.056 g, 1.0 mmol) were dissolved in chloroform (4 ml). To the solution was added 1-naphthaldehyde (0.094 g, 0.6 mmol) at 298 K, the solution was stirred for 6 h and removal of solvent under reduced pressure, the residue was purified through column chromatography. Colourless single crystals of (I) were obtained by recrystallization from an ethyl acetate solution.

### Refinement

Imine H atom was located in a difference Fourier map and refined isotropically, with restrains of N—H = 0.84±1 Å. The carbon-bound hydrogen atoms were placed in calculated positions with C—H = 0.93–0.98 Å, and refined using a riding model with *U*_iso_(H) =1.2*U*_eq_(C). In the absence of significant anomalous scattering effects, Friedel pairs were merged.

### Crystal data

**Table:**

C_19_H_15_NO_2_	*F*(000) = 608
*M**_r_* = 289.32	*D*_x_ = 1.245 Mg m^−^^3^
Orthorhombic, *P*2_1_2_1_2_1_	Cu *K*α radiation, λ = 1.54184 Å
Hall symbol: P 2ac 2ab	Cell parameters from 9381 reflections
*a* = 6.6289 (1) Å	θ = 3.8–72.1°
*b* = 10.0350 (1) Å	µ = 0.65 mm^−^^1^
*c* = 23.2033 (3) Å	*T* = 290 K
*V* = 1543.51 (3) Å^3^	Block, colorless
*Z* = 4	0.40 × 0.36 × 0.30 mm

### Data collection

**Table:**

Oxford Diffraction Gemini S Ultra diffractometer	1783 independent reflections
Radiation source: Enhance Ultra (Cu) X-ray Source	1641 reflections with *I* > 2σ(*I*)
mirror	*R*_int_ = 0.031
Detector resolution: 15.9149 pixels mm^-1^	θ_max_ = 72.3°, θ_min_ = 3.8°
ω scans	*h* = −6→8
Absorption correction: multi-scan (*CrysAlis PRO*; Oxford Diffraction, 2009)	*k* = −12→12
*T*_min_ = 0.782, *T*_max_ = 0.829	*l* = −26→28
13761 measured reflections	

### Refinement

**Table:**

Refinement on *F*^2^	Primary atom site location: structure-invariant direct methods
Least-squares matrix: full	Secondary atom site location: difference Fourier map
*R*[*F*^2^ > 2σ(*F*^2^)] = 0.032	Hydrogen site location: inferred from neighbouring sites
*wR*(*F*^2^) = 0.093	H atoms treated by a mixture of independent and constrained refinement
*S* = 1.08	*w* = 1/[σ^2^(*F*_o_^2^) + (0.0641*P*)^2^ + 0.0314*P*] where *P* = (*F*_o_^2^ + 2*F*_c_^2^)/3
1783 reflections	(Δ/σ)_max_ < 0.001
203 parameters	Δρ_max_ = 0.08 e Å^−^^3^
0 restraints	Δρ_min_ = −0.13 e Å^−^^3^

### Special details

**Table:**

Geometry. All e.s.d.'s (except the e.s.d. in the dihedral angle between two l.s. planes) are estimated using the full covariance matrix. The cell e.s.d.'s are taken into account individually in the estimation of e.s.d.'s in distances, angles and torsion angles; correlations between e.s.d.'s in cell parameters are only used when they are defined by crystal symmetry. An approximate (isotropic) treatment of cell e.s.d.'s is used for estimating e.s.d.'s involving l.s. planes.
Refinement. Refinement of *F*^2^ against ALL reflections. The weighted *R*-factor *wR* and goodness of fit *S* are based on *F*^2^, conventional *R*-factors *R* are based on *F*, with *F* set to zero for negative *F*^2^. The threshold expression of *F*^2^ > σ(*F*^2^) is used only for calculating *R*-factors(gt) *etc*. and is not relevant to the choice of reflections for refinement. *R*-factors based on *F*^2^ are statistically about twice as large as those based on *F*, and *R*- factors based on ALL data will be even larger.

### Fractional atomic coordinates and isotropic or equivalent isotropic displacement parameters (Å^2^)

**Table:**

	*x*	*y*	*z*	*U*_iso_*/*U*_eq_	
O1	1.20270 (19)	0.10810 (12)	0.82723 (5)	0.0681 (3)	
O2	1.0096 (2)	0.38001 (11)	0.74225 (7)	0.0738 (4)	
N1	0.8951 (2)	0.16688 (13)	0.74912 (6)	0.0534 (3)	
H4	0.926 (3)	0.092 (2)	0.7622 (8)	0.065 (5)*	
C1	0.3564 (3)	0.1625 (3)	0.65510 (8)	0.0820 (6)	
H1	0.2376	0.1592	0.6339	0.098*	
C2	0.4104 (3)	0.0578 (2)	0.68938 (9)	0.0750 (5)	
H2	0.3275	−0.0168	0.6917	0.090*	
C3	0.5874 (3)	0.06219 (17)	0.72067 (8)	0.0619 (4)	
H3	0.6224	−0.0090	0.7443	0.074*	
C4	0.7135 (2)	0.17226 (15)	0.71700 (6)	0.0521 (3)	
C5	0.6590 (3)	0.27941 (19)	0.68282 (8)	0.0659 (4)	
H5	0.7411	0.3543	0.6804	0.079*	
C6	0.4786 (3)	0.2728 (2)	0.65216 (9)	0.0806 (6)	
H6	0.4402	0.3445	0.6292	0.097*	
C7	1.0258 (2)	0.26612 (14)	0.75961 (7)	0.0523 (3)	
C8	1.2046 (2)	0.23071 (15)	0.79612 (7)	0.0558 (4)	
H8	1.3359	0.2573	0.7805	0.067*	
C9	1.1896 (2)	0.23005 (17)	0.85940 (8)	0.0597 (4)	
H9	1.3135	0.2555	0.8797	0.072*	
C10	0.9999 (3)	0.25565 (18)	0.89182 (7)	0.0588 (4)	
C11	0.8539 (3)	0.1600 (2)	0.89662 (8)	0.0738 (5)	
H11	0.8689	0.0791	0.8776	0.089*	
C12	0.6808 (3)	0.1835 (3)	0.93030 (9)	0.0855 (7)	
H12	0.5819	0.1181	0.9330	0.103*	
C13	0.6567 (3)	0.2994 (3)	0.95855 (9)	0.0854 (7)	
H13	0.5414	0.3127	0.9807	0.103*	
C14	0.8028 (3)	0.4013 (2)	0.95534 (7)	0.0718 (5)	
C15	0.9780 (3)	0.38002 (19)	0.92061 (7)	0.0587 (4)	
C16	1.1212 (3)	0.4825 (2)	0.91701 (8)	0.0690 (4)	
H16	1.2345	0.4710	0.8939	0.083*	
C17	1.0979 (4)	0.5988 (3)	0.94673 (10)	0.0880 (6)	
H17	1.1938	0.6659	0.9432	0.106*	
C18	0.9322 (5)	0.6176 (3)	0.98222 (11)	0.1012 (8)	
H18	0.9205	0.6956	1.0036	0.121*	
C19	0.7874 (4)	0.5231 (3)	0.98595 (9)	0.0927 (7)	
H19	0.6752	0.5383	1.0091	0.111*	

### Atomic displacement parameters (Å^2^)

**Table:**

	*U*^11^	*U*^22^	*U*^33^	*U*^12^	*U*^13^	*U*^23^
O1	0.0674 (7)	0.0523 (6)	0.0846 (7)	0.0119 (6)	−0.0146 (6)	−0.0057 (5)
O2	0.0694 (7)	0.0412 (6)	0.1109 (10)	−0.0041 (6)	−0.0195 (7)	0.0016 (6)
N1	0.0506 (7)	0.0396 (6)	0.0700 (7)	−0.0001 (5)	−0.0054 (6)	−0.0003 (6)
C1	0.0604 (10)	0.1084 (17)	0.0771 (11)	0.0011 (11)	−0.0134 (9)	−0.0229 (12)
C2	0.0523 (9)	0.0768 (12)	0.0960 (13)	−0.0071 (9)	0.0031 (9)	−0.0250 (11)
C3	0.0518 (8)	0.0524 (8)	0.0814 (10)	−0.0007 (7)	0.0049 (7)	−0.0099 (8)
C4	0.0486 (8)	0.0488 (7)	0.0590 (8)	0.0025 (7)	0.0020 (6)	−0.0085 (6)
C5	0.0660 (10)	0.0609 (9)	0.0707 (9)	−0.0014 (8)	−0.0075 (8)	0.0042 (8)
C6	0.0768 (12)	0.0928 (14)	0.0722 (11)	0.0095 (12)	−0.0161 (10)	0.0058 (10)
C7	0.0491 (8)	0.0411 (7)	0.0666 (8)	0.0019 (6)	−0.0003 (7)	−0.0092 (6)
C8	0.0455 (7)	0.0464 (7)	0.0754 (9)	0.0041 (7)	−0.0016 (7)	−0.0099 (7)
C9	0.0476 (8)	0.0591 (8)	0.0725 (9)	0.0031 (7)	−0.0106 (7)	−0.0094 (7)
C10	0.0505 (8)	0.0687 (9)	0.0571 (8)	0.0000 (8)	−0.0086 (7)	0.0031 (7)
C11	0.0653 (10)	0.0803 (12)	0.0757 (10)	−0.0129 (10)	−0.0124 (9)	0.0083 (10)
C12	0.0612 (11)	0.1093 (17)	0.0859 (13)	−0.0194 (13)	−0.0057 (10)	0.0263 (13)
C13	0.0564 (11)	0.131 (2)	0.0693 (10)	0.0054 (12)	0.0082 (9)	0.0238 (12)
C14	0.0595 (10)	0.1020 (14)	0.0539 (8)	0.0149 (11)	0.0018 (7)	0.0095 (9)
C15	0.0531 (8)	0.0723 (10)	0.0505 (7)	0.0054 (8)	−0.0034 (6)	0.0030 (7)
C16	0.0660 (10)	0.0751 (11)	0.0658 (9)	−0.0003 (9)	−0.0002 (8)	−0.0080 (9)
C17	0.0988 (16)	0.0799 (13)	0.0853 (12)	−0.0038 (13)	−0.0017 (12)	−0.0181 (11)
C18	0.120 (2)	0.1003 (17)	0.0831 (13)	0.0244 (18)	0.0033 (14)	−0.0278 (13)
C19	0.0897 (15)	0.1226 (19)	0.0658 (10)	0.0355 (16)	0.0118 (11)	−0.0050 (12)

### Geometric parameters (Å, °)

**Table:**

O1—C8	1.4266 (19)	C9—C10	1.488 (2)
O1—C9	1.436 (2)	C9—H9	0.9800
O2—C7	1.217 (2)	C10—C11	1.367 (3)
N1—C7	1.342 (2)	C10—C15	1.423 (2)
N1—C4	1.417 (2)	C11—C12	1.408 (3)
N1—H4	0.84 (2)	C11—H11	0.9300
C1—C2	1.365 (3)	C12—C13	1.345 (4)
C1—C6	1.373 (3)	C12—H12	0.9300
C1—H1	0.9300	C13—C14	1.410 (3)
C2—C3	1.381 (3)	C13—H13	0.9300
C2—H2	0.9300	C14—C19	1.417 (4)
C3—C4	1.388 (2)	C14—C15	1.429 (2)
C3—H3	0.9300	C15—C16	1.402 (3)
C4—C5	1.384 (2)	C16—C17	1.364 (3)
C5—C6	1.393 (3)	C16—H16	0.9300
C5—H5	0.9300	C17—C18	1.386 (4)
C6—H6	0.9300	C17—H17	0.9300
C7—C8	1.499 (2)	C18—C19	1.352 (4)
C8—C9	1.472 (2)	C18—H18	0.9300
C8—H8	0.9800	C19—H19	0.9300
			
C8—O1—C9	61.87 (10)	O1—C9—H9	114.9
C7—N1—C4	127.99 (14)	C8—C9—H9	114.9
C7—N1—H4	116.1 (15)	C10—C9—H9	114.9
C4—N1—H4	115.8 (15)	C11—C10—C15	120.32 (16)
C2—C1—C6	119.65 (18)	C11—C10—C9	121.23 (17)
C2—C1—H1	120.2	C15—C10—C9	118.36 (15)
C6—C1—H1	120.2	C10—C11—C12	120.3 (2)
C1—C2—C3	120.29 (19)	C10—C11—H11	119.8
C1—C2—H2	119.9	C12—C11—H11	119.8
C3—C2—H2	119.9	C13—C12—C11	120.8 (2)
C2—C3—C4	120.33 (18)	C13—C12—H12	119.6
C2—C3—H3	119.8	C11—C12—H12	119.6
C4—C3—H3	119.8	C12—C13—C14	121.31 (19)
C5—C4—C3	119.75 (15)	C12—C13—H13	119.3
C5—C4—N1	123.56 (15)	C14—C13—H13	119.3
C3—C4—N1	116.68 (14)	C13—C14—C19	123.3 (2)
C4—C5—C6	118.67 (18)	C13—C14—C15	118.65 (19)
C4—C5—H5	120.7	C19—C14—C15	118.0 (2)
C6—C5—H5	120.7	C16—C15—C10	123.11 (16)
C1—C6—C5	121.3 (2)	C16—C15—C14	118.30 (18)
C1—C6—H6	119.4	C10—C15—C14	118.59 (17)
C5—C6—H6	119.4	C17—C16—C15	121.4 (2)
O2—C7—N1	125.45 (15)	C17—C16—H16	119.3
O2—C7—C8	118.66 (14)	C15—C16—H16	119.3
N1—C7—C8	115.89 (13)	C16—C17—C18	120.5 (2)
O1—C8—C9	59.38 (10)	C16—C17—H17	119.8
O1—C8—C7	118.91 (13)	C18—C17—H17	119.8
C9—C8—C7	120.76 (13)	C19—C18—C17	120.4 (2)
O1—C8—H8	115.4	C19—C18—H18	119.8
C9—C8—H8	115.4	C17—C18—H18	119.8
C7—C8—H8	115.4	C18—C19—C14	121.4 (2)
O1—C9—C8	58.75 (10)	C18—C19—H19	119.3
O1—C9—C10	117.46 (15)	C14—C19—H19	119.3
C8—C9—C10	124.10 (14)		
			
C6—C1—C2—C3	−0.4 (3)	O1—C9—C10—C15	−175.59 (13)
C1—C2—C3—C4	−0.8 (3)	C8—C9—C10—C15	−106.34 (18)
C2—C3—C4—C5	1.5 (2)	C15—C10—C11—C12	−0.3 (3)
C2—C3—C4—N1	−178.11 (15)	C9—C10—C11—C12	176.26 (16)
C7—N1—C4—C5	11.2 (2)	C10—C11—C12—C13	−0.5 (3)
C7—N1—C4—C3	−169.22 (16)	C11—C12—C13—C14	0.2 (3)
C3—C4—C5—C6	−0.9 (3)	C12—C13—C14—C19	−177.7 (2)
N1—C4—C5—C6	178.69 (16)	C12—C13—C14—C15	0.8 (3)
C2—C1—C6—C5	1.0 (3)	C11—C10—C15—C16	−179.42 (17)
C4—C5—C6—C1	−0.4 (3)	C9—C10—C15—C16	4.0 (2)
C4—N1—C7—O2	−0.7 (3)	C11—C10—C15—C14	1.2 (2)
C4—N1—C7—C8	179.01 (13)	C9—C10—C15—C14	−175.40 (14)
C9—O1—C8—C7	−110.62 (15)	C13—C14—C15—C16	179.15 (17)
O2—C7—C8—O1	165.96 (15)	C19—C14—C15—C16	−2.3 (2)
N1—C7—C8—O1	−13.7 (2)	C13—C14—C15—C10	−1.5 (2)
O2—C7—C8—C9	96.3 (2)	C19—C14—C15—C10	177.07 (16)
N1—C7—C8—C9	−83.36 (18)	C10—C15—C16—C17	−177.89 (18)
C8—O1—C9—C10	115.07 (16)	C14—C15—C16—C17	1.5 (3)
C7—C8—C9—O1	107.55 (15)	C15—C16—C17—C18	1.1 (4)
O1—C8—C9—C10	−103.91 (18)	C16—C17—C18—C19	−2.7 (4)
C7—C8—C9—C10	3.6 (2)	C17—C18—C19—C14	1.8 (4)
O1—C9—C10—C11	7.8 (2)	C13—C14—C19—C18	179.2 (2)
C8—C9—C10—C11	77.1 (2)	C15—C14—C19—C18	0.7 (3)

### Hydrogen-bond geometry (Å, °)

**Table:**

*D*—H···*A*	*D*—H	H···*A*	*D*···*A*	*D*—H···*A*
N1—H4···O2^i^	0.84 (2)	2.17 (2)	2.954 (1)	155.7 (18)
C5—H5···O1^ii^	0.93	2.58	3.431 (2)	153 (1)

Symmetry codes: (i) −*x*+2, *y*−1/2, −*z*+3/2; (ii) −*x*+2, *y*+1/2, −*z*+3/2.
